# Supplementary material for: Iterative computational design and crystallographic screening identifies potent inhibitors targeting the Nsp3 Macrodomain of SARS-CoV-2
Source: bioRxiv. 2022 Jul 28:2022.06.27.497816. Originally published 2022 Jun 28. Preprint. [Version 2] doi: 10.1101/2022.06.27.497816 (PMC9258288; doi:10.1101/2022.06.27.497816)
Supplement: Supplement 6 [file media-6.pdf]

Dataset S5: Crystal structures of Mac1 in complex with analogs of docking hits (LL1 series). PanDDA event maps are shown for ligands (contoured at 2 σ). Protein-ligand hydrogen bonds are shown with dashed black lines. Hydrogen bonds between ligands and the Lys11 backbone nitrogen of a symmetry mate are highlighted with purple spheres/dashes.

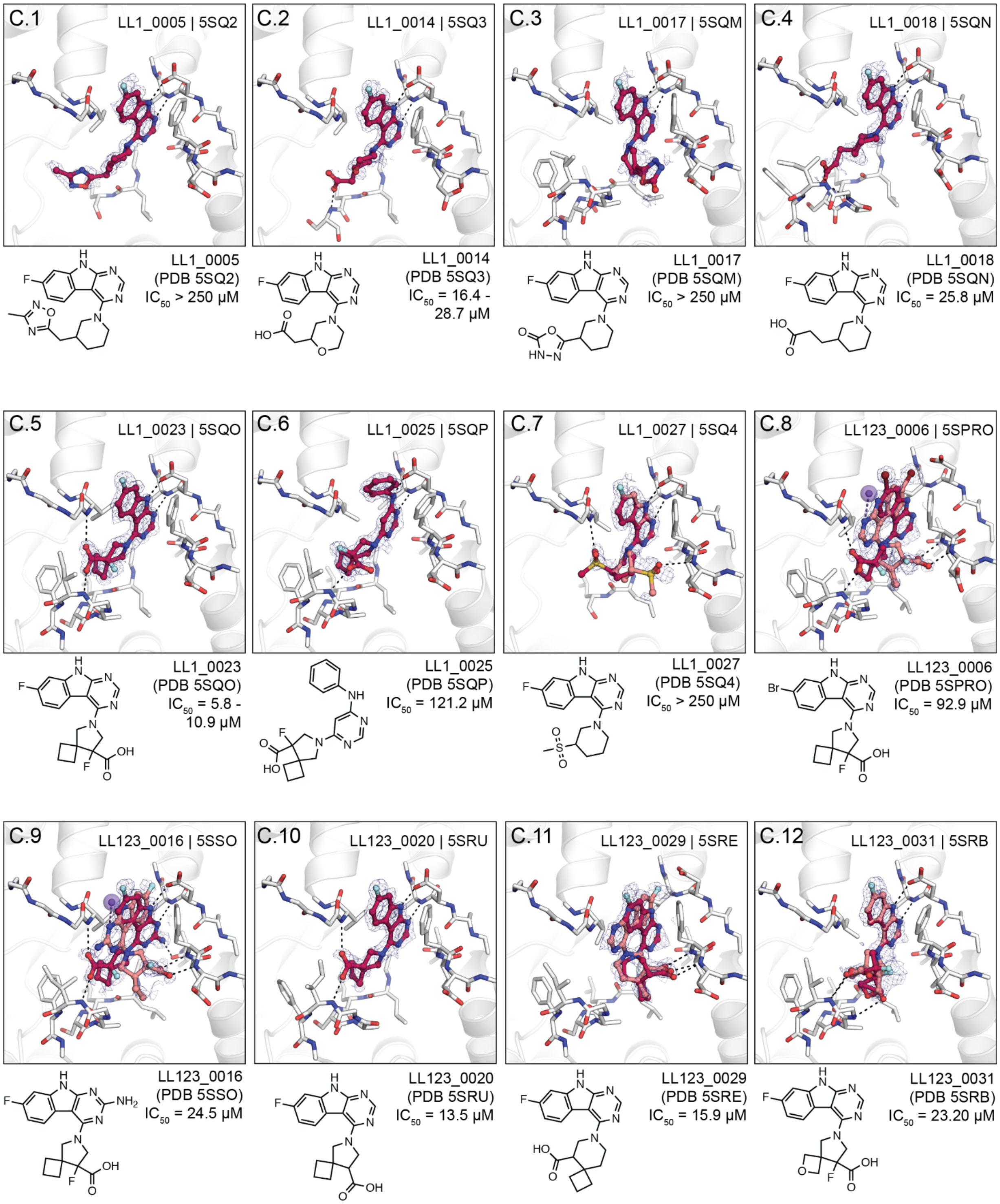

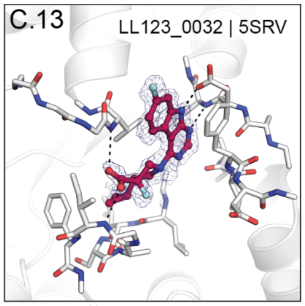

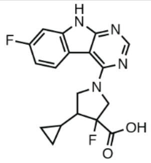

LL123_0032
(PDB 5SRV)
IC_{50} = 41.8 µM

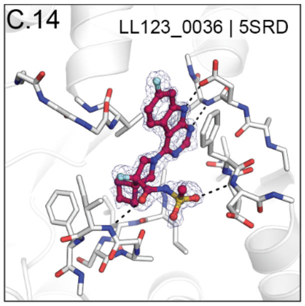

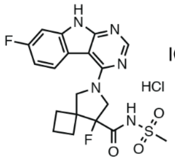

LL123_0036
(PDB 5RSD)
IC_{50} = 17.1 µM

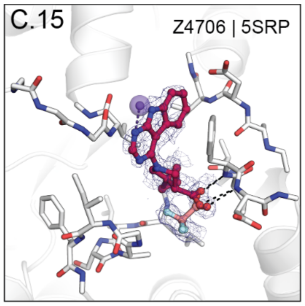

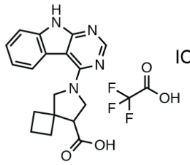

Z4706
(PDB 5SRP)
IC_{50} = 15.4 µM

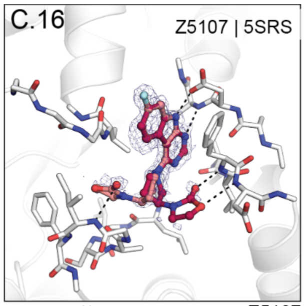

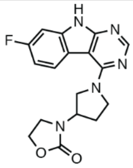

Z5107
(PDB 5SRS)
IC_{50} > 200 µM

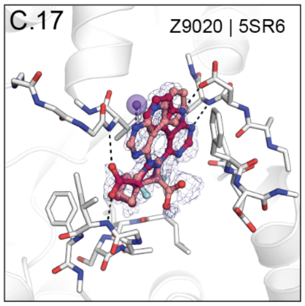

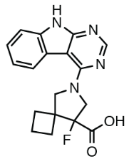

Z9020
(PDB 5SR6)
IC_{50} = 36.8 µM

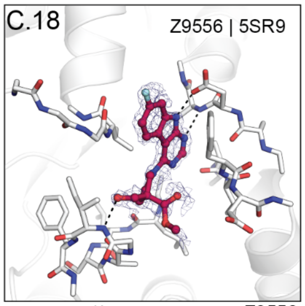

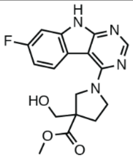

Z9556
(PDB 5SR9)
IC_{50} > 200 µM

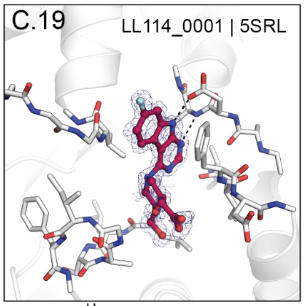

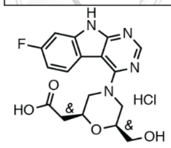

LL114_0001
(PDB 5SRL)
IC_{50} = 8.2 µM

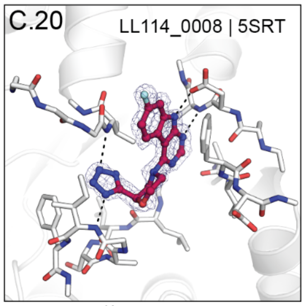

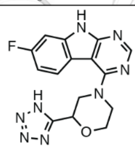

LL114_0008
(PDB 5SRT)
IC_{50} = 17.2 µM

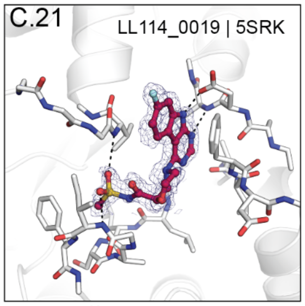

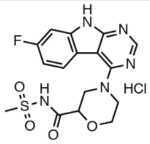

LL114_0019
(PDB 5SRK)
IC_{50} = 12.3 µM

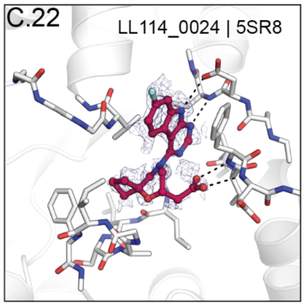

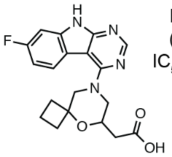

LL114_0024
(PDB 5SR8)
IC_{50} = 19.6 µM

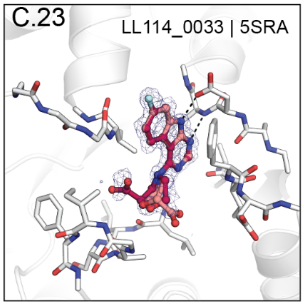

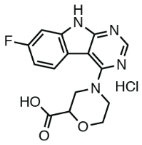

LL114_0033
(PDB 5SRA)
IC_{50} = 40.4 µM

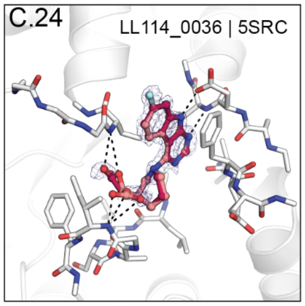

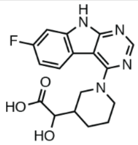

LL114_0036
(PDB 5SRC)
IC_{50} = 34.1 µM

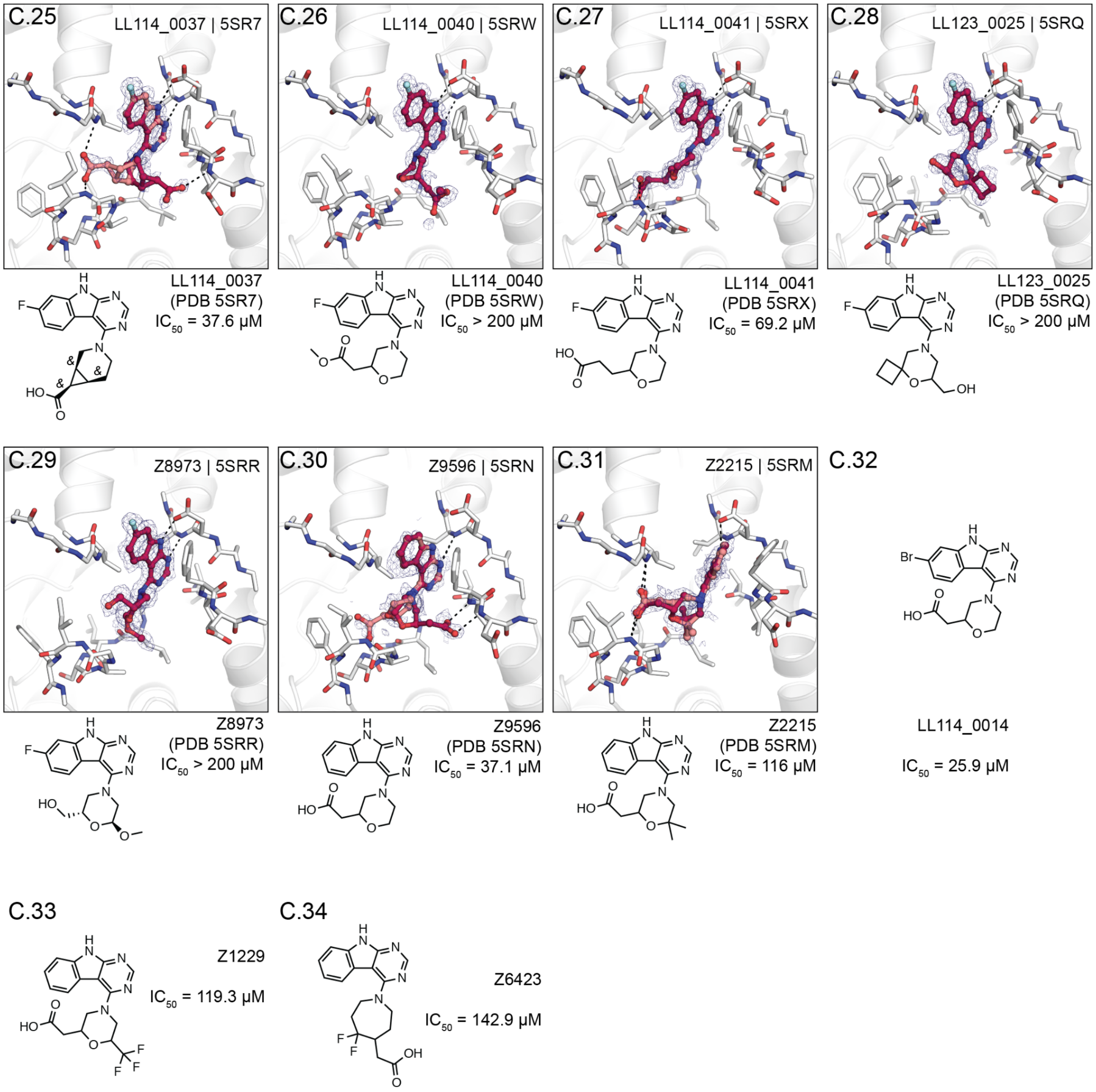
C.25
LL114_0037 | 5SR7
LL114_0037
(PDB 5SR7)
IC50 = 37.6 μM
C.26
LL114_0040 | 5SRW
LL114_0040
(PDB 5SRW)
IC50 > 200 μM
C.27
LL114_0041 | 5SRX
LL114_0041
(PDB 5SRX)
IC50 = 69.2 μM
C.28
LL123_0025 | 5SRQ
LL123_0025
(PDB 5SRQ)
IC50 > 200 μM
C.29
Z8973 | 5SRR
Z8973
(PDB 5SRR)
IC50 > 200 μM
C.30
Z9596 | 5SRN
Z9596
(PDB 5SRN)
IC50 = 37.1 μM
C.31
Z2215 | 5SRM
Z2215
(PDB 5SRM)
IC50 = 116 μM
C.32
LL114_0014
IC50 = 25.9 μM
C.33
Z1229
IC50 = 119.3 μM
C.34
Z6423
IC50 = 142.9 μM
