# Supplementary material for: Iterative computational design and crystallographic screening identifies potent inhibitors targeting the Nsp3 Macrodomain of SARS-CoV-2
Source: bioRxiv. 2022 Jul 28:2022.06.27.497816. Originally published 2022 Jun 28. Preprint. [Version 2] doi: 10.1101/2022.06.27.497816 (PMC9258288; doi:10.1101/2022.06.27.497816)
Supplement: Supplement 7 [file media-7.pdf]

Dataset S6: Design of neutral probe set. Two carboxylic acids were included as close analogs. Crystal structures of Mac1 in complex with neutral probe set hits. PanDDA event maps are shown for ligands (contoured at 2 σ). Protein-ligand hydrogen bonds are shown with dashed black lines. Hydrogen bonds between ligands and the Lys11 backbone nitrogen of a symmetry mate are highlighted with purple spheres/dashes.

D.1

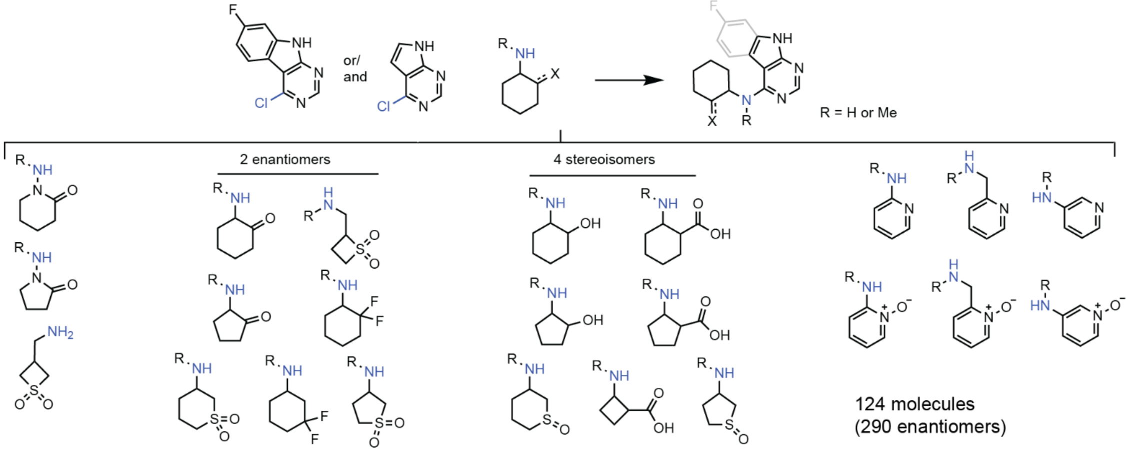

D.2

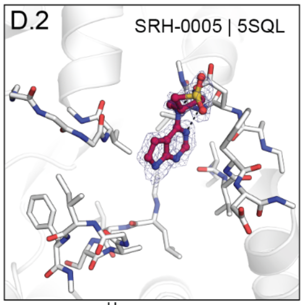

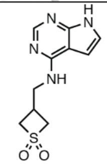

SRH-0005
(PDB 5SQL)
IC_{50} > 200 µM

D.3

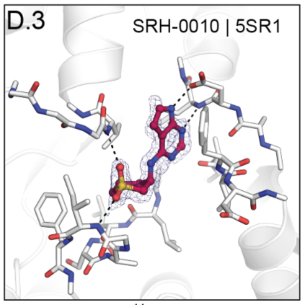

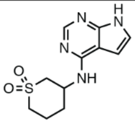

SRH-0010
(PDB 5SR1)
IC_{50} > 200 µM

D.4

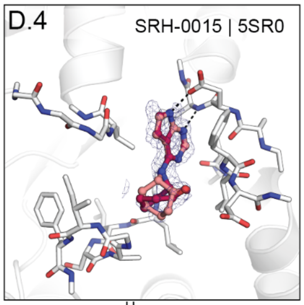

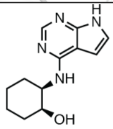

SRH-0015
(PDB 5SR0)
IC_{50} = 131.6 µM

D.5

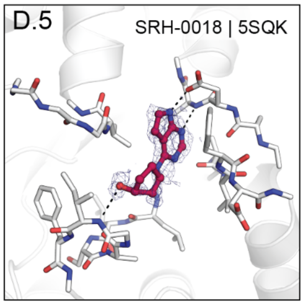

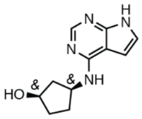

SRH-0018
(PDB 5SQK)
IC_{50} > 200 µM

D.6

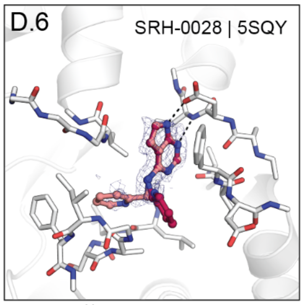

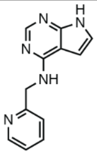

SRH-0028
(PDB 5SQY)
IC_{50} > 200 µM

D.7

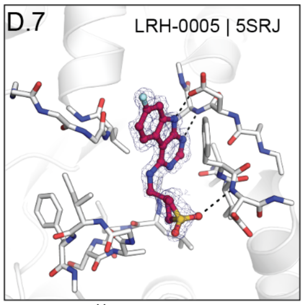

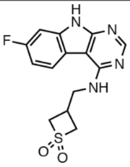

LRH-0005
(PDB 5SRJ)
IC_{50} > 200 µM

D.8

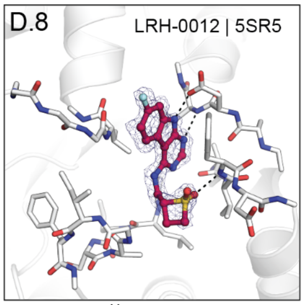

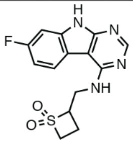

LRH-0012
(PDB 5SR5)
IC_{50} > 200 µM

D.9

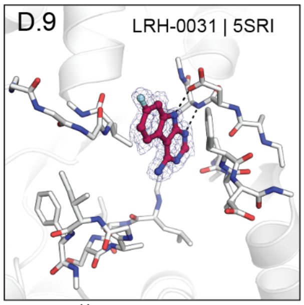

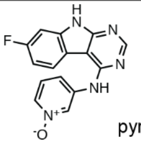

LRH-0031
(PDB 5SRI)
IC_{50} > 200 µM

no desnity for
pyridin-1-ium-1-olate

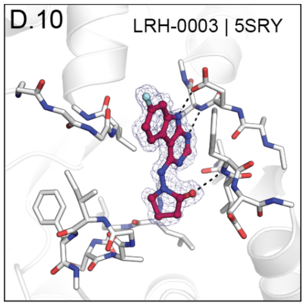

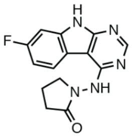

LRH-0003
(PDB 5SRY)
IC_{50} = 1.7 μM

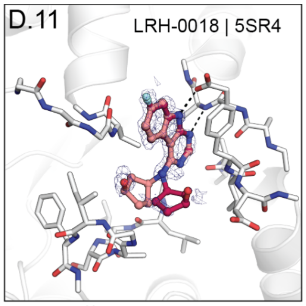

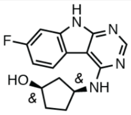

LRH-0018
(PDB 5SR4)
IC_{50} > 200 μM

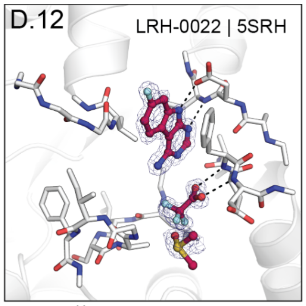

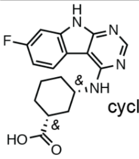

LRH-0022
(PDB 5SRH)
IC_{50} > 200 μM
no density for
cyclohexanecarboxylic
acid

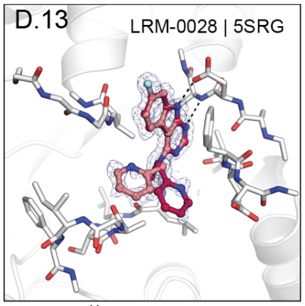

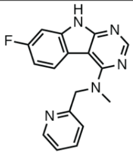

LRM-0028
(PDB 5SRG)
IC_{50} > 200 μM

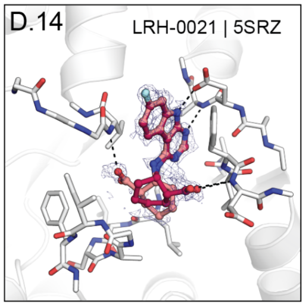

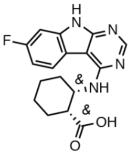

LRH-0021
(PDB 5SRZ)
IC_{50} = 1.7 μM

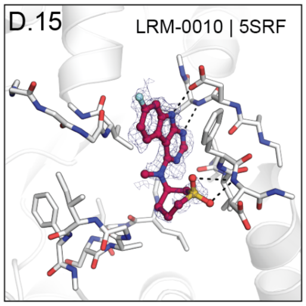

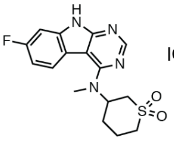

LRM-0010
(PDB 5SRF)
IC_{50} > 200 μM

D.16

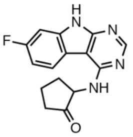

LRH-0008

IC_{50} = 13.4 μM
