# Supplementary material for: Structure-based inhibitor optimization for the Nsp3 Macrodomain of SARS-CoV-2
Source: bioRxiv. 2022 Jun 28:2022.06.27.497816. Preprint. [Version 1] doi: 10.1101/2022.06.27.497816 (PMC9258288; doi:10.1101/2022.06.27.497816)
Supplement: Supplementary file 4 [file media-6.pdf]

Data file S5: Crystal structures of Mac1 in complex with analogs of docking hits (LL1 series). PanDDA event maps are shown for ligands (contoured at 2 σ). Protein-ligand hydrogen bonds are shown with dashed black lines. Hydrogen bonds between ligands and the Lys11 backbone nitrogen of a symmetry mate are highlighted with purple spheres/dashes.

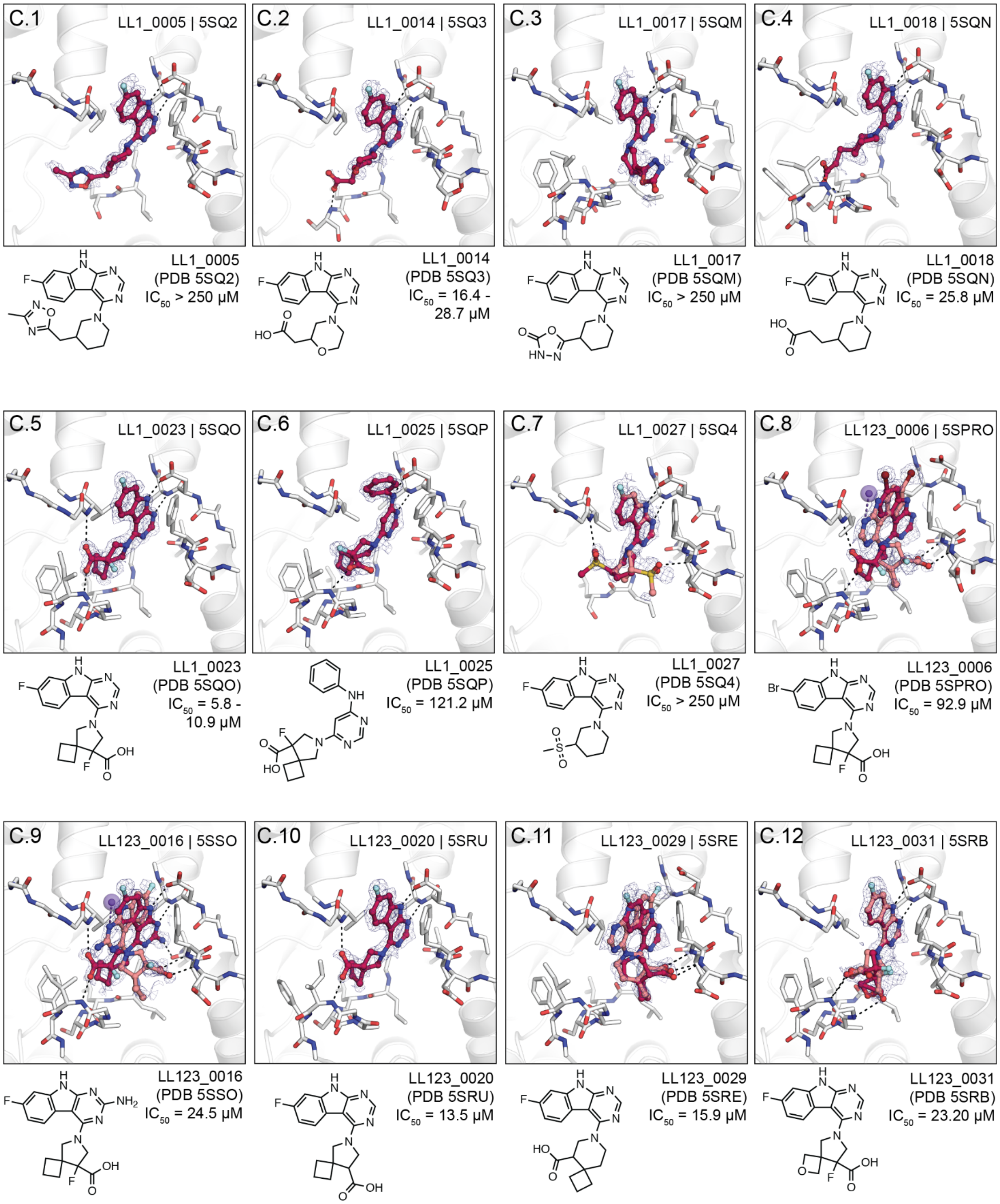

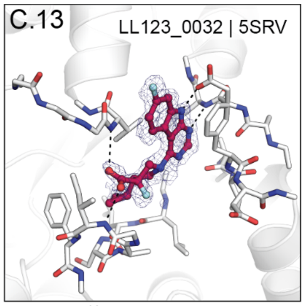

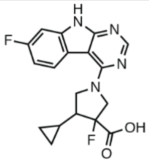

LL123_0032
(PDB 5SRV)
IC_{50} = 41.8 μM

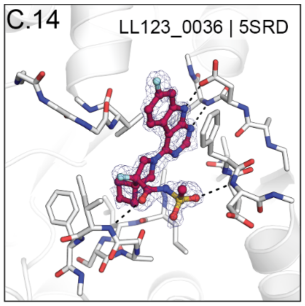

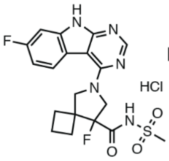

LL123_0036
(PDB 5RSD)
IC_{50} = 17.1 μM

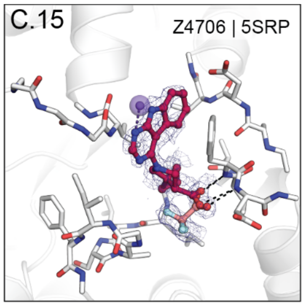

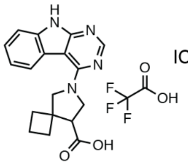

Z4706
(PDB 5SRP)
IC_{50} = 15.4 μM

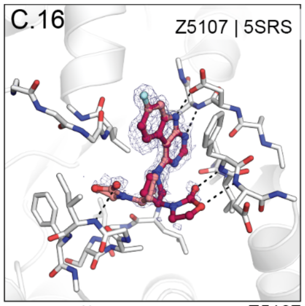

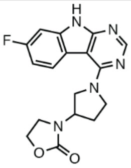

Z5107
(PDB 5SRS)
IC_{50} > 200 μM

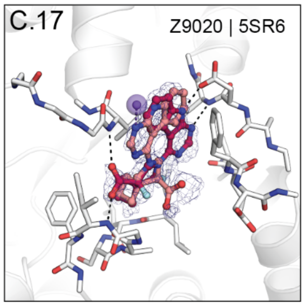

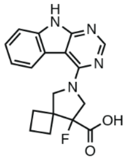

Z9020
(PDB 5SR6)
IC_{50} = 36.8 μM

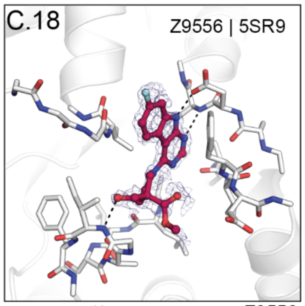

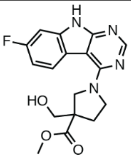

Z9556
(PDB 5SR9)
IC_{50} > 200 μM

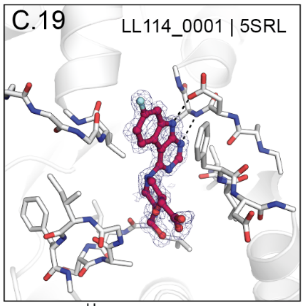

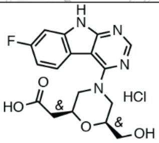

LL114_0001
(PDB 5SRL)
IC_{50} = 8.2 μM

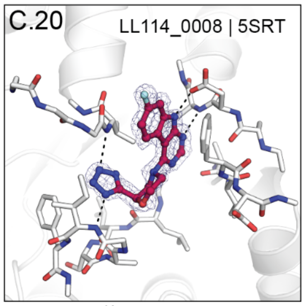

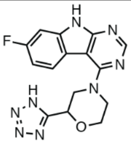

LL114_0008
(PDB 5SRT)
IC_{50} = 17.2 μM

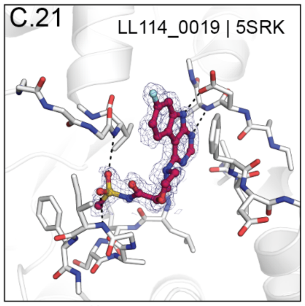

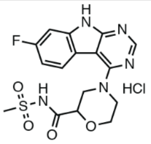

LL114_0019
(PDB 5SRK)
IC_{50} = 12.3 μM

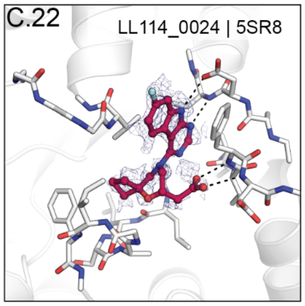

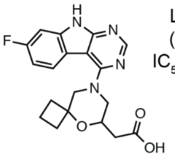

LL114_0024
(PDB 5SR8)
IC_{50} = 19.6 μM

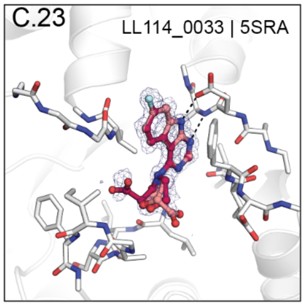

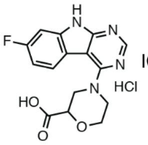

LL114_0033
(PDB 5SRA)
IC_{50} = 40.4 μM

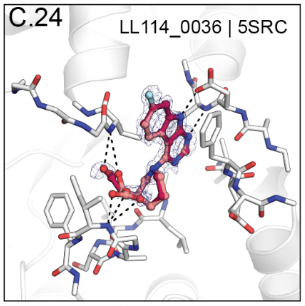

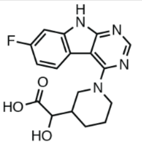

LL114_0036
(PDB 5SRC)
IC_{50} = 34.1 μM

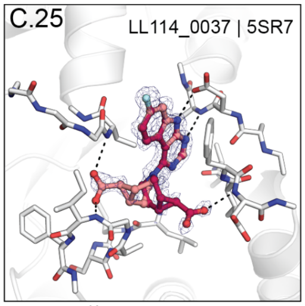

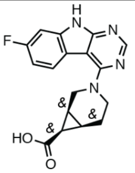

LL114_0037
(PDB 5SR7)
IC_{50} = 37.6 µM

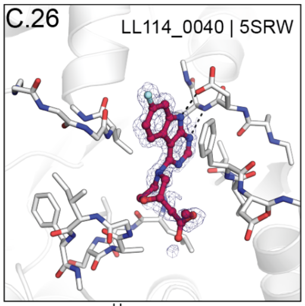

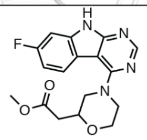

LL114_0040
(PDB 5SRW)
IC_{50} > 200 µM

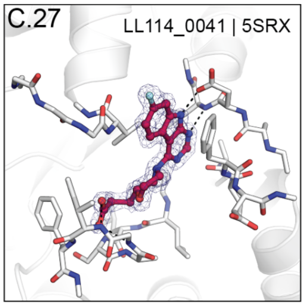

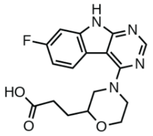

LL114_0041
(PDB 5SRX)
IC_{50} = 69.2 µM

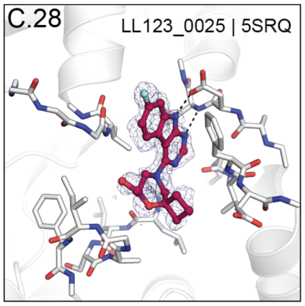

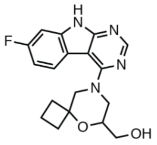

LL123_0025
(PDB 5SRQ)
IC_{50} > 200 µM

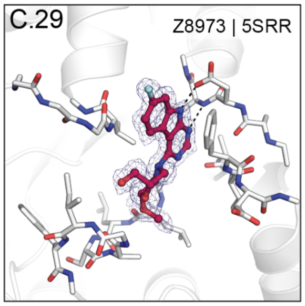

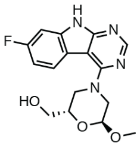

Z8973
(PDB 5SRR)
IC_{50} > 200 µM

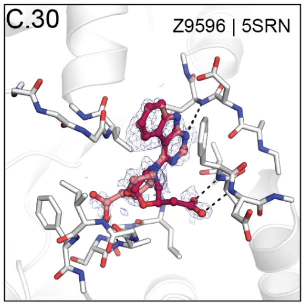

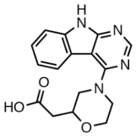

Z9596
(PDB 5SRN)
IC_{50} = 37.1 µM

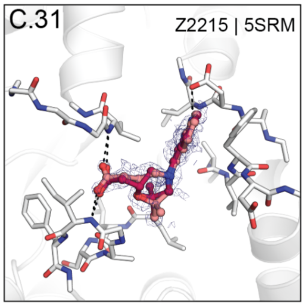

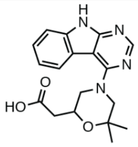

Z2215
(PDB 5SRM)
IC_{50} = 116 µM

C.32

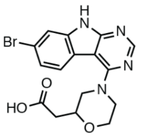

LL114_0014

IC_{50} = 25.9 µM

C.33

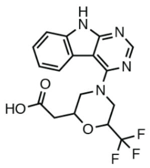

Z1229

IC_{50} = 119.3 µM

C.34

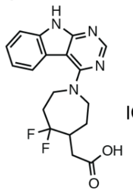

Z6423

IC_{50} = 142.9 µM
