# Supplementary material for: Structure-based inhibitor optimization for the Nsp3 Macrodomain of SARS-CoV-2
Source: bioRxiv. 2022 Jun 28:2022.06.27.497816. Preprint. [Version 1] doi: 10.1101/2022.06.27.497816 (PMC9258288; doi:10.1101/2022.06.27.497816)
Supplement: Supplementary file 5 [file media-7.pdf]

Data file S6: Design of neutral probe set. Two carboxylic acids were included as close analogs. Crystal structures of Mac1 in complex with neutral probe set hits. PanDDA event maps are shown for ligands (contoured at 2 σ). Protein-ligand hydrogen bonds are shown with dashed black lines. Hydrogen bonds between ligands and the Lys11 backbone nitrogen of a symmetry mate are highlighted with purple spheres/dashes.

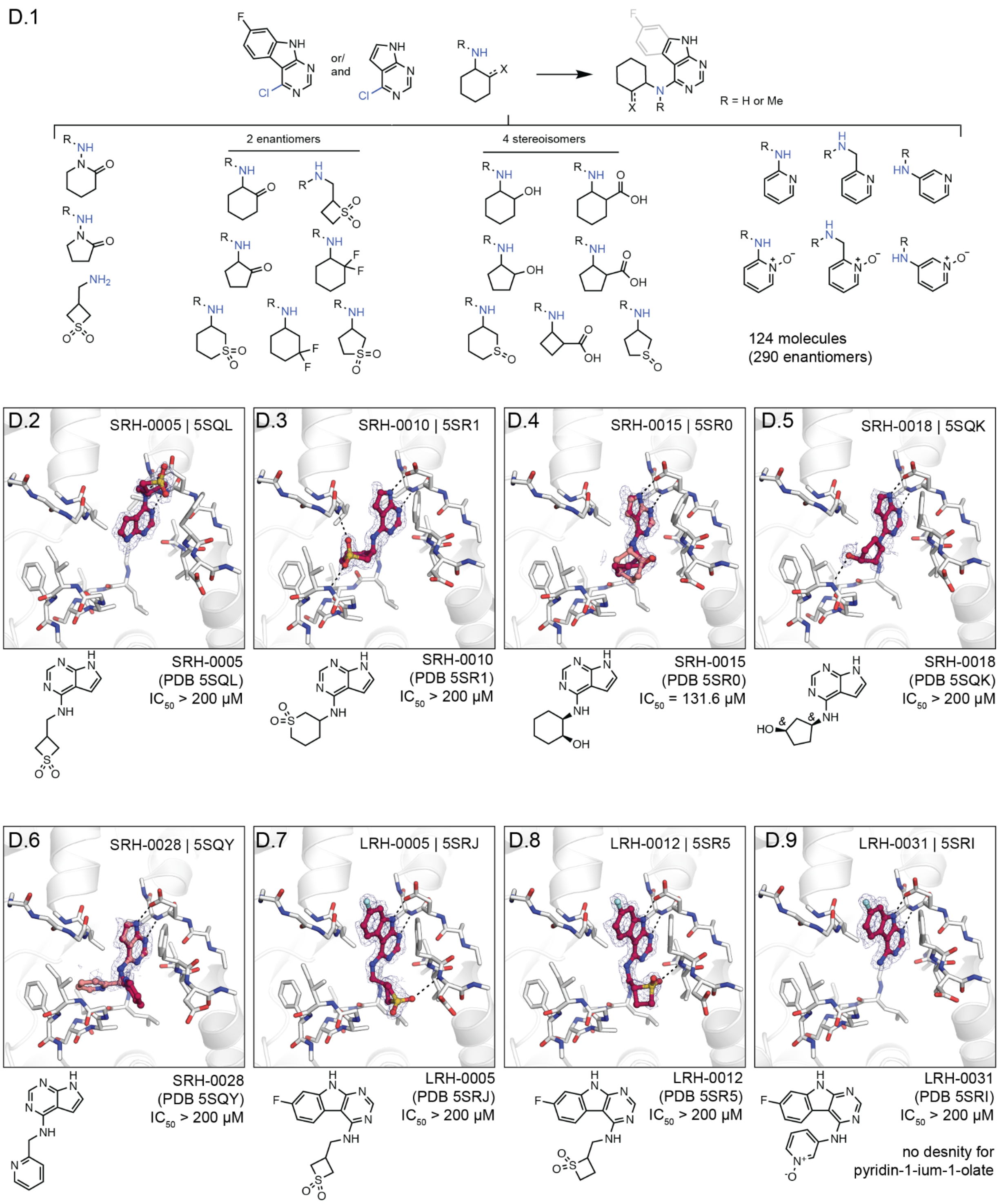

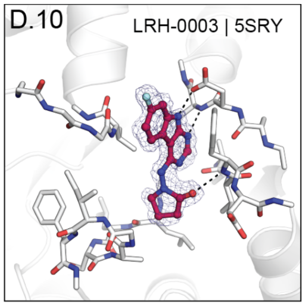

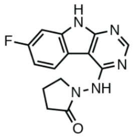

LRH-0003
(PDB 5SRY)
IC_{50} = 1.7 µM

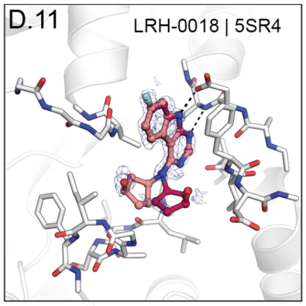

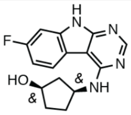

LRH-0018
(PDB 5SR4)
IC_{50} > 200 µM

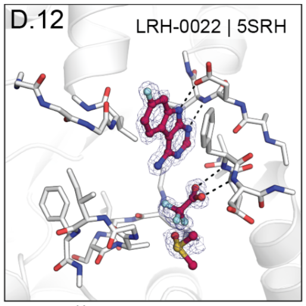

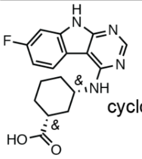

LRH-0022
(PDB 5SRH)
IC_{50} > 200 µM
no density for
cyclohexanecarboxylic
acid

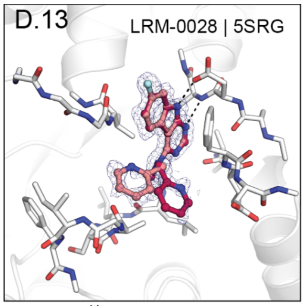

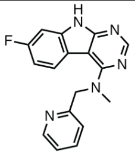

LRM-0028
(PDB 5SRG)
IC_{50} > 200 µM

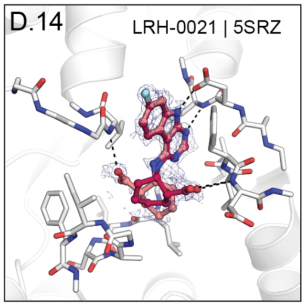

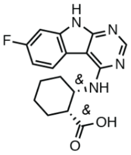

LRH-0021
(PDB 5SRZ)
IC_{50} = 1.7 µM

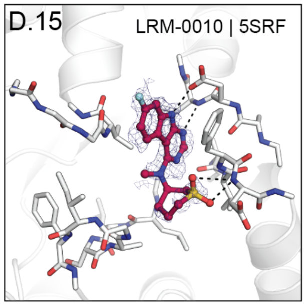

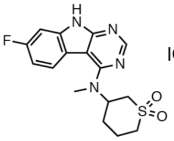

LRM-0010
(PDB 5SRF)
IC_{50} > 200 µM

D.16

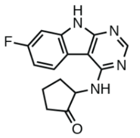

LRH-0008

IC_{50} = 13.4 µM
